# Synthesis and Characterization of Compounds Related to Lisinopril

**DOI:** 10.3797/scipharm.1507-08

**Published:** 2015-10-18

**Authors:** Ambati V. Raghava Reddy, Srinivas Garaga, Chandiran Takshinamoorthy, Andra Naidu, Ramesh Dandala

**Affiliations:** 1 Chemical Research and Development Department, Aurobindo Pharma Ltd, Survey No:71&72, Indrakaran Village, Sangareddy Mandal, Medak district, Telangana, 502329, India; 2 Department of Chemistry, Jawaharlal Nehru Technological University Hyderabad, Kukatpally, Hyderabad, Telangana State, 500085, India; 3 Affliated supervisor, Jawaharlal Nehru Technological University Hyderabad, Kukatpally, Hyderabad, Telangana State, 500085, India

**Keywords:** Lisinopril, Related substances, Origin, Synthesis, Characterization, Impurities

## Abstract

Lisinopril is a drug of the angiotensin-converting enzyme (ACE) inhibitor class that is primarily used in the treatment of hypertension. During the scale-up of the lisinopril process, one unknown impurity was observed and is identified. The present work describes the origin, synthesis, characterization, and control of this impurity. This paper also describes the synthesis and characterization of three other impurities listed in the European Pharmacopoeia 8.4 (Impurity C, D, and F).

## Introduction

Lisinopril is the third ACE inhibitor after captopril and enalapril, and is chemically known as *N*^2^-[(1*S*)-1-Carboxy-3-phenylpropyl]-L-lysyl-L-proline. Lisinopril is marketed by Merck under the brand name of PRINVIL^®^.

**Fig. 1:**
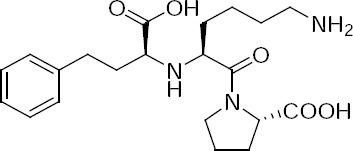
Structure of lisinopril.

The presence of impurities in an Active Pharmaceutical Ingredient (API) will influence the quality and safety of the drug product. Hence, an impurity profile study has to be carried out for any drug substance and it is also a regulatory requirement to identify and characterize all unknown impurities. Further, impurities are required in pure form to check the analytical performance characteristics such as specificity, linearity, range, accuracy, precision, limit of detection (LOD), limit of quantification (LOQ), robustness, system suitability testing, and relative retention factor [1,2].

During the scale-up of the lisinopril process, one unknown impurity was observed at a level of 0.1–0.2% long with other impurities listed in the European Pharmacopoeia [3]. The unknown impurity detected was monitored and its structure was tentatively assigned on the basis of its fragmentation patterns in the LC-MS analysis. Further, this impurity was synthesized, characterized, and co-injected with lisinopril in HPLC analysis to confirm its structure. Based on the structural data, the chemical name of this impurity is 2-(1-(5-amino-1-carboxypentylcarbamoyl)-5-aminopentylamino)-4-phenylbutanoic acid (*N*^2^-(1-Carboxy-3-phenylpropyl)lysyllysine, lysine analogue). The present work also describes the synthesis and characterization of three other known impurities by various spectroscopic techniques.

## Results and Discussion

Lisinopril **1** has been synthesized by known literature methods [4,5]. Our route of synthesis for lisinopril is shown in Scheme 1. *L*-lysine (**2**) was reacted with ethyltrifluoro acetate to obtain *N*^6^-trifluoroacetyl-*L*-lysine (**3**). Compound **3** was treated with triphosgene to obtain *N*^6^-trifluoroacetyl-*N*^2^-carboxy-*L*-lysine anhydride (**4**), which was condensed with *L*-proline to get *N*^6^-trifluoroacetyl-*L*-lysyl-*L*-proline (**5**). Compound **5** was condensed with ethyl 2-oxo-4-phenyl butyrate followed by hydrogenation in the presence of the Raney-Nickel catalyst to get *N*^2^-(1-(*S*)-ethoxycarbonyl-3-phenylpropyl)-*L*-*N*^6^-(trifluoroacetyl)-*L*-lysyl-*L*-proline (**6**). Finally, compound **6** was hydrolyzed with sodium hydroxide which resulted in lisinopril **1**.

**Sch. 1:**
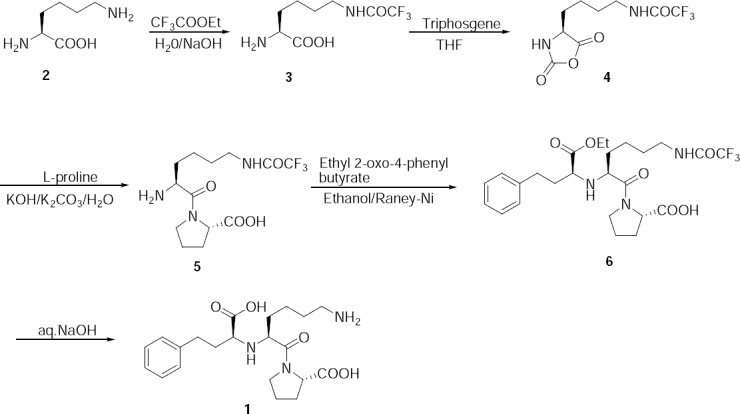
Reported synthetic route for lisinopril.

This paper reports the synthesis and characterization of the unknown impurity (lysine analogue) along with three known impurities [6–18].

**Fig. 2:**
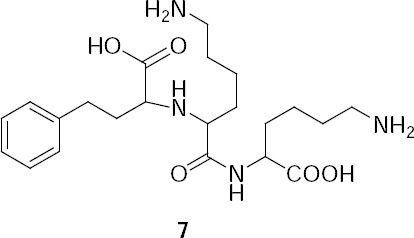
Chemical structure of the lysine analogue 7.

The chemical structures of the impurities listed in the European Pharmacopoeia 8.4 are given below: (*S,S,S*)-diketopiperazine (**8**), (*R,S,S*)-diketopiperazine (**9**), and cyclohexyl analogue (**10**).

**Fig. 3:**
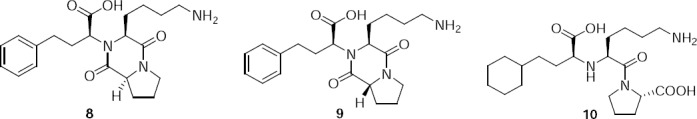
Structures of lisinopril impurities.

The chemical names of the impurities **7–10** are given below.

*N*^2^-(1-Carboxy-3-phenylpropyl)lysyllysine (**7**, lysine analogue).

(2*S*)-2-[(3*S*,8a*S*)-3-(4-Aminobutyl)-1,4-dioxohexahydropyrrolo[1,2-*a*]pyrazin-2(1*H*)-yl]-4-phenylbutanoic acid [**8**, (*S,S,S*)-Diketopiperazine, Ph. Eur Impurity-C)].

(2*S*)-2-[(3*S*,8*aR*)-3-(4-Aminobutyl)-1,4-dioxohexahydropyrrolo[1,2-*a*]pyrazin-2(1*H*)-yl]-4-phenylbutanoic acid [**9**, (*R,S,S*)-Diketopiperazine, Ph. Eur Impurity-D)].

1-{*N*^2^-[(1*S*)-1-Carboxy-3-cyclohexylpropyl]-L-lysyl}-1*H*-pyrrole-2-carboxylic acid [**10**, (Cyclohexyl Analogue, Ph. Eur Impurity-F)].

The origin, synthesis, characterization, and control of these related substances are discussed individually. Each of the synthesized impurities was characterized by conventional spectroscopic studies and the presences of these impurities were confirmed by co-injection with the lisinopril sample. To the best of our knowledge, the synthesis of these related substances were not yet reported.

### Route Cause for the Formation of Lisinopril Lysine Analogue (7)

Lysine analogue **7** formed due to the presence of unreacted protected lysine **3** in *N*^6^-trifluoroacetyl-*N*^2^-carboxy-*L*-lysine anhydride (**4**). This unreacted compound **3** underwent all the reactions employed for the synthesis of lisinopril to give lysine analogue **7**.

**Sch. 2:**
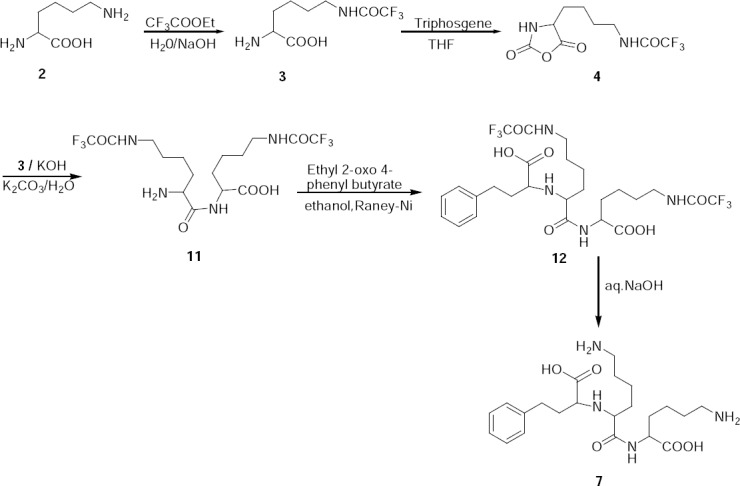
Synthesis of lysine analogue 7.

Lysine analogue **7** was independently prepared by the condensation of compound **3** with **4** in the presence of potassium hydroxide and potassium carbonate to give condensed product **11**. Compound **11** was reacted with ethyl 2-oxo-4-phenyl butyrate to form a Schiff base, which upon catalytic hydrogenation in the presence of Raney-Nickel, produced compound **12**. Finally, compound **12** was hydrolysed to give lisinopril lysine analogue (**7**). The mass spectrum showed a molecular ion at m/z 437.3 amu [(M+H)^+^]. The ^1^H-NMR spectrum of **7** showed the presence of two –NH_2_ and two –NH groups which confirmed the assigned structure.

### Route Cause for the Formation of (S,S,S)-diketopiperazine (Ph. Eur Impurity-C, 8)

(*S,S,S*)-Diketopiperazine is the one of the listed impurities in lisinopril. This impurity may be formed due to intramolecular dehydration of lisinopril at high temperature.

(*S,S,S*)-Diketopiperazine was independently prepared by heating the n-butanol solution of lisinopril in the presence of hydrochloric acid at 80°C. The spectral data of this impurity matched the reported results [16].

**Sch. 3:**
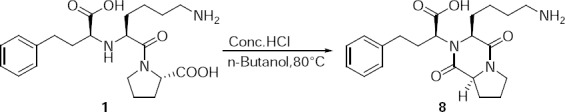
Synthesis of (S,S,S)-diketopiperazine.

### Route Cause for the Formation of (R,S,S)-diketopiperazine (Ph. Eur Impurity-D, 9)

**(*R,S,S*)-**Diketopiperazine is one of the known impurities in lisinopril. This impurity may be formed due to epimerization of Impurity-C at high temperature.

**(*R,S,S*)-**Diketopiperazine was independently prepared by heating lisinopril at 190°C. The spectral data of this impurity matched the reported results [16].

**Sch. 4:**
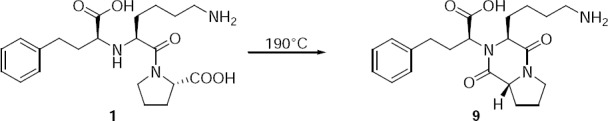
Synthesis of (*R,S,S*)-diketopiperazine.

### Route Cause for the Formation of the Cyclohexyl Analogue (Ph. Eur Impurity-F)

The cyclohexyl analogue is one of the known impurities in lisinopril. This impurity is formed due to the over-hydrogenation at the condensation reaction of compound **5** with ethyl 2-oxo-4-phenyl butyrate in the presence of Raney-Ni and H_2_ pressure. This impurity can be controlled by maintaining the controlled temperature and hydrogen pressure.

This cyclohexyl analogue was independently prepared by hydrogenation of lisinopril **1** with the Rodium catalyst in methanol. The mass spectrum showed a molecular ion at m/z 411 amu [(M+H)^+^]. The ^1^H-NMR spectrum of **10** showed the absence of aromatic protons, confirming the assigned structure.

**Sch. 5:**
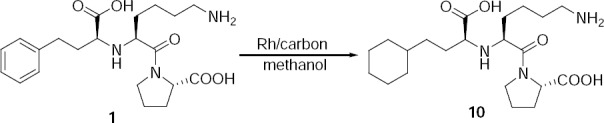
Synthesis of the cyclohexyl analogue 10.

## Experimental

Solvents and reagents were obtained from commercial sources and used without purification. ^1^H-NMR and ^13^C-NMR spectral data were performed on the Bruker-Avance 300-MHz Spectrometer in DMSO-d_6_ and D_2_O wherever applicable. The chemical shift values were reported on the δ scale in parts per million (*ppm*), downfield from tetramethylsilane (TMS) as an internal standard. The IR spectra were recorded in the solid state as KBr pellets using a Perkin-Elmer FT-IR Spectrophotometer. The mass spectrum was recorded using a Perkin-Elmer PE SCIEX-API 2000, equipped with an ESI source used online with an HPLC system after the ultraviolet (UV) detector.

### N^2^-(1-Carboxy-3-phenylpropyl)lysyllysine (7, lysine analogue) [2-(1-(5-amino)-1-carboxypentylcarbamoyl)-5-aminopentylamino)-4-phenylbutanoic acid, 6-Amino-2-({6-amino-2-[(1-carboxy-3-phenylpropyl)amino]hexanoyl}amino)hexanoic acid]

#### Synthesis of compound 4

To a solution of triphosgene (25.8 g, 86.86 mmol) in tetrahydofuran (250 mL), compound **3** (50 g, 206.6 mmol) was added at 25–30°C. The reaction mixture was heated to 35–40°C and maintained for 20 h. After completion of the reaction, we concentrated the reaction mass at 35–40°C under reduced pressure to obtain an oily mass **4**. (Note: this oily material was taken as such for the next step without characterization).

#### Synthesis of compound 11

To a mixture of potassium hydroxide (30.62 g, 546.78 mmol), potassium carbonate (42.83 g, 310.36 mmol) in DM water (700 mL), and tetrahydrofuran (500 mL), was added protected lysine **3** (50 g, 206.6 mmol) at 0–5°C followed by compound **4**. Thereafter, the reaction mass was stirred vigorously for 20 mins to complete the reaction. We adjusted the pH of the reaction mass to ~5 with sulfuric acid at 0–5°C and then concentrated it under reduced pressure at 35–40°C to obtain a residue. This residue was dissolved in methanol (200 mL) and we continued distillation to remove water completely. Again methanol (200 mL) was added to the resulting syrupy mass and it was stirred for 20 mins at 25–30°C. Inorganic salts thus precipitated were filtered and the filtrate was concentrated under reduced pressure at 35–40°C. The resulting residue was titurated with MTBE to obtain **11** as a white solid (72 g, 75%).

^1^H-NMR (DMSO-D_6_, 300 MHz): 1.29–1.41 (m, 12H), 4.02 (m, 2H), 8.57 (brs, NH), 9.50 (brs, NH) *ppm*, *m/z*: 466.8 [(M+H)^+^].

#### Synthesis of compound 7

In one litre, a hydrogenator charged a solution of compound **11** (25 g, 53.64 mmol, in 400 mL methanol), and to it was added molecular sieves powder (50 g, type 3A) followed by ethyl-2-oxo-4-phenyl butyrate (13.81 g, 67 mmol) and Raney-Ni (25 g). Continued hydrogenation was done at 25–30°C with 4 kg/cm^2^ hydrogen pressure for 16 h. The reaction was monitored by TLC and after completion of the reaction, the mass was carefully filtered under nitrogen atmosphere to remove the Raney-Nickel catalyst. The methanol filtrate was concentrated and the residue obtained was dissolved in DM water (125 mL). We adjusted the pH to 8.0 with 5% w/w aqueous sodium bicarbonate solution and then washed it with toluene (125 mL). Dichloromethane (125 mL) was added to the aqueous layer and we adjusted the pH of the reaction mixture to 4.5 with concentrated hydrochloric acid. The organic layer was separated and concentrated under reduced pressure at 35-40°C, resulting in compound **12** as a residue.

HPLC Purity: 96.54%; ^1^H-NMR (DMSO-D_6_, 300 MHz): 1.16 (m, 3H), 1.16–1.19 (m, 4H), 1.21–1.28 (m, 6H), 1.42–1.49 (m, 4H), 2.50–2.51 (m, 2H), 2.92–3.35 (m, 6H), 4.07–4.10 (q, 2H), 4.11 (m, 1H), 7.14–7.29 (m, 5H), 7.90–7.93 (d, 1H) *ppm*; MS *m/z*: 657.1 [(M+H)+].

DM water (200 mL) was added to residue **12** and pH was adjusted to 12.5 with 50% w/w aqueous sodium hydroxide solution. The reaction mass was stirred for 3 h at 40-45°C by maintaining pH at 12-12.5 with 50% w/w aqueous sodium hydroxide solution. The reaction mass was cooled to 20-25°C and pH was adjusted to 5.0 with concentrated hydrochloric acid. The reaction mass was concentrated under reduced pressure at 35-40°C. The residue was dissolved in methanol and concentrated at 35-40°C to ensure the complete removal of water. Again, methanol (50 mL) was added to the residue and stirred for 20 mins. The resulting inorganic salts were removed by filtration and the filtrate was concentrated under reduced pressure at 35-40°C. The residue obtained was crystallized from acetone (50 mL) to obtain lysine analogue **7** (20 g, 83%).

HPLC Purity: 96.16%; IR (KBr pellet, cm^−1^): 3434 (OH str), 3089 (Ar CH str) 2959–2870 (aliphatic CH str), 1640 (C=O str), 1454 (CH_2_ bend); ^1^H-NMR (D_2_O, 300 MHz): 1.31–2.19 (m, 14H), 2.76 (m, 2H), 2.91-2.98 (m, 4H), 3.36 & 3.98 (2m, 2H), 4.33 (m, 1H), 7.28–7.37 (m, 5H, Ar) *ppm*; ^13^C-NMR (D_2_O, 75 MHz): 178.5, 174.9, 171.1, 138.7, 129.17, 128.95, 126.96, 61.1, 60.47, 53.78, 45.8, 45.3, 42.1, 32.9, 32.5, 32.1, 30.7, 28, 21, 21.1 *ppm*; MS *m/z*: 437.3 [(M+H)+].

### (2S)-2-[(3S,8aS)-3-(4-Aminobutyl)-1,4-dioxohexahydropyrrolo[1,2-a]pyrazin-2(1H)-yl]-4-phenylbutanoic acid [8, (S,S,S)-Diketopiperazine, Ph. Eur Impurity-C]

To a suspension of lisinopril dihydrate (50 g, 113.37 mmol) in n-butanol (500 mL) was added concentrated hydrochloric acid (12.9 g) at 25-30°C. The reaction mixture was heated to 75–80°C and stirred for 24 h. The reaction was cooled to 25–30°C and pH was adjusted to 5.2 with 5 N sodium hydroxide. Thereafter, the reaction mixture was concentrated under reduced pressure to a syrupy mass. To the resulting syrupy mass was added n-butanol (100 mL) and the salts were removed by filtration. The filtrate was concentrated under reduced pressure at 50–55°C resulting in an oily mass. The resulting oily mass was purified by column chromatography by using ethyl acetate and methanol to obtain (*S,S,S*)-diketopiperazine (20 g, 40%).

HPLC Purity: 90.48%; IR (KBr pellet, cm^−1^): 3200-3600 (OH, NH str), 3000-2800 (CH_2_), 1650 (C=O str), 1587 (asymmetric CO_2_ str), 1387 (symmetric CO_2_ str), 755,702 (aromatic CH out of plane); ^1^H-NMR (D_2_O, 300 MHz): 0.77–0.87 (m, 1H), 1.44–2.07 (m, 9H), 2.33-2.84 (m, 6H), 3.10–3.50 (m, 3H), 3.76 (dd, 1H), 3.85 (brs, 1H), 7.10–7.27 (m, 5H, Ar) *ppm*; MS *m/z*: 387 [(M+H)+].

### (2S)-2-[(3S,8aR)-3-(4-Aminobutyl)-1,4-dioxohexahydropyrrolo[1,2-a]pyrazin-2(1H)-yl]-4-phenylbutanoic acid [9, (R,S,S)-Diketopiperazine, Ph. Eur Impurity-D)

Lisinopril dihydrate (100 g, 226.7 mmol) was placed in an RB flask and heated to about 190°C with a continuous flow of nitrogen for 4 h. The resulting molten residue was slowly cooled to 25–30°C and further purified through column chromatography by using ethyl acetate and a hexane mixture to obtain 50 g (58%) of compound **9**.

HPLC Purity: 89.29%; IR (KBr pellet, cm^−1^): 3200–3600 (OH, NH str), 3000–2800 (CH_2_), 1650 (C=O str), 1587 (asymmetric CO_2_ str), 1387 (symmetric CO_2_ str), 755, 702 (aromatic CH out of plane); ^1^H-NMR (D_2_O, 300 MHz): 1.17–1.22 (m, 1H), 1.40–2.11 (m, 9H), 2.33–2.86 (m, 6H), 3.66 (t, 1H), 3.80 (dd, 1H), 4.10 (dd, 1H), 7.10–7.23 (m, 5H, Ar) *ppm*; MS *m/z*: 387 [(M+H)+].

### 1-{N^2^-[(1S)-1-Carboxy-3-cyclohexylpropyl]-L-lysyl}-1H-pyrrole-2-carboxylic acid 1-[(2S)-6-Amino-2-{[(1S)-1-carboxy-3-cyclohexylpropyl]amino}hexanoyl]-1H-pyrrole-2-carboxylic acid (10, Cyclohexyl Analogue, Ph. Eur Impurity-F)

To a solution of lisinopril dihydrate (5 g, 11.33 mmol) in DM water (5 mL) and methanol (50 mL) was added rhodium on carbon (0.4 g) at 25–30°C. The reaction mixture was charged into the hydrogenator and 15 kg/cm^2^ hydrogen pressure was applied at 25–30°C. The reaction mass was stirred for 20 h at 25–30°C by maintaining hydrogen pressure at 15 kg/cm^2^. Then reaction mixture was filtered and washed with methanol (10 mL) and concentrated under reduced pressure at 35–40°C, resulting in a residue. The resulting residue was purified by column chromatography resulting in the desired product **10** (3.3 g, 60%).

HPLC Purity: 94.6%; IR (KBr pellet, cm^−1^): 3200–3600 (OH, NH str), 2924–2851 (CH_2_), 1644 (C=O str), 1449 (CH_2_ bending), 1391 (symmetric CO_2_ str), 755, 702 (aromatic CH out of plane); ^1^H-NMR (D_2_O, 300 MHz): 0.78–2.10 (m, 24H), 2.19–2.23 (m, 1H), 2.95 (t, 2H), 3.31 (t, 1H), 3.44–3.55 (m, 2H), 3.76–4.28 (m, 2H) *ppm*; MS *m/z*:411 [(M+H)+].
